# Relationship between miR-203a inhibition and oil-induced toxicity in early life stage zebrafish (*Danio rerio*)

**DOI:** 10.1016/j.toxrep.2022.03.006

**Published:** 2022-03-07

**Authors:** Jason T. Magnuson, Le Qian, Victoria McGruer, Vanessa Cheng, David C. Volz, Daniel Schlenk

**Affiliations:** a Department of Environmental Sciences, University of California, Riverside, CA, USA; b College of Sciences, China Agricultural University, Beijing, China; c College of Horticulture and Plant Protection, Henan University of Science and Technology, Luoyang, China; d Institute of Environmental Health, College of Environmental and Resource Sciences, Zhejiang University, Hangzhou 310058, China

**Keywords:** MicroRNA, Early life stage, Zebrafish, Fin development, Optokinetic response assay

## Abstract

Dysregulation of microRNA (miRNA, miR) by environmental stressors influences the transcription of mRNA which may impair organism development and/or lead to adverse physiological outcomes. Early studies evaluating the effects of oil on developmental toxicity in early life stages of fish showed that reductions in expression of miR-203a were associated with enhanced expression of downstream mRNAs that predicted altered eye development, cardiovascular disease, and improper fin development. To better understand the effects of miR-203a inhibition as an outcome of oil-induced toxicity in early life stage (ELS) fish, embryonic zebrafish were injected with an miR-203a inhibitor or treated with 3.5 µM phenanthrene (Phe) as a positive control for morphological alterations of cardiovascular and eye development caused by oil. Embryos treated with Phe had diminished levels of miR-203a at 7 and 72 h after injection. Embryos treated with the miR-203a inhibitor and Phe exhibited a reduced heart rate by 48 h post fertilization (hpf), with an increased incidence of developmental deformities (including pericardial edema, altered eye development, and spinal deformities) and reduced caudal fin length by 72 hpf. There were significant reductions in lens and eye diameters in 120 hpf miR-203a-inhibitor and Phe-treated fish, as well as a significantly reduced number of eye saccades, determined by an optokinetic response (OKR) behavioral assay. The expression of *vegfa*, which is an important activator during neovascularization, was significantly upregulated in embryos receiving miR-203a inhibitor injections by 7 and 72 hpf with increased trends in *vegfa* expression in 72 hpf larvae treated with Phe. There were decreasing trends in *crx*, *neurod1*, and *pde6h* expression by 72 hpf in miR-203a inhibitor and Phe treatments, which are involved in photoreceptor function in developing eyes and regulated by miR-203a. These results suggest that an inhibition of miR-203a in ELS fish exhibits an oil-induced toxic response that is consistent with Phe treatment and specifically impacts retinal, cardiac, and fin development in ELS fish.

## Highlights

• miR-203a inhibitor-injected zebrafish exhibited an oil-induced toxic response.
• Inhibition of miR-203a impaired retinal, cardiac, and fin development in zebrafish.
• miR-203a inhibition validated previously predicted transcriptomic pathways.

## Graphical Abstract

**Figure:**
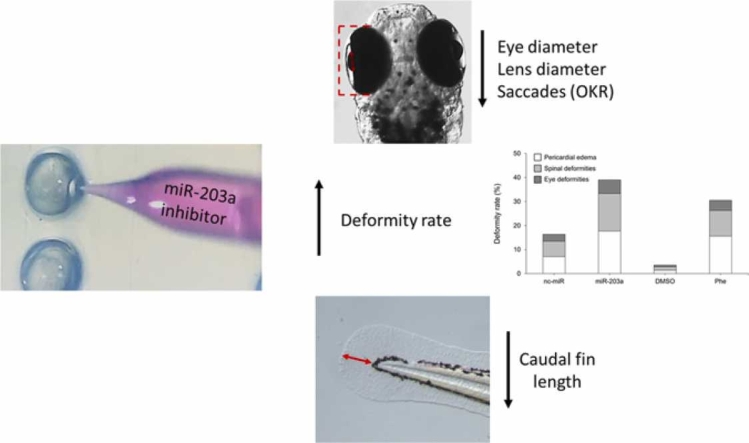

## 1 Introduction

Polycyclic aromatic hydrocarbons (PAHs) are a ubiquitous class of compounds that are comprised of 2–8 fused benzene rings that can be introduced into aquatic systems naturally, through oil seeps, or anthropogenic actions, such as through the incomplete combustion of fossil fuels and oil spills [1], [2]. PAHs (parent, alkylated, and hydroxylated confirmations) are the primary toxic constituents in crude oil and are well known to induce developmental defects in early life stage (ELS) fish, which is among the most sensitive life stage to PAHs following exposure. Tricyclic PAHs, such as phenanthrene (Phe), comprised the largest fraction of measured PAHs in weathered oil collected from the surface following the *Deepwater Horizon* (DWH) oil spill in 2010 [3]. As such, PAHs present in DWH oil have been related to an increased incidence of craniofacial deformities, cardiotoxicity, and impaired eye development and visual function in ELS fish [4], [5], [6], [7], [8], [9], [10].

The use of transcriptomics and bioinformatic predictions has allowed for subsequent, hypothesis-driven research to characterize mechanistic relationships between mRNA and microRNA (miRNA; miR) to disease and function pathways in fish exposed to crude oil [12], [10], [11]. miRNAs are small (~22 nucleotides), non-coding RNAs that regulate post-transcriptional mRNA expression [13]. Understanding how dysregulation of miRNA influence mRNA transcription following exposure to environmental contaminants is increasing in importance as more is understood about the role of miRNAs in development and disease [15], [16], [14], [17], [18], [19]. ELS mahi-mahi (*Coryphaena hippurus*) [10] and red drum (*Sciaenops ocellatus*) [12] exposed to crude oil indicated consistent, differentially expressed miRNAs following treatment to weathered slick and source oil. Of the consistently dysregulated miRNAs, inhibition of miR-203a expression overlapped with potential downstream genes involved in contributing to neurological, cardiovascular, and ophthalmic disease and skeletal/muscular disorders [12], [10].

miR-203a is an essential regulator of genes responsible for proper retina, cardiac, and fin development, function, and regeneration [21], [22], [23], [24], [25], [20]. Altered miR-203a expression has been associated with inducing myocardial fibrosis in cultured mouse cardiomyocytes [26], regulating progenitor cell proliferation in the retina of zebrafish (*Danio rerio*) [23], and caudal fin regeneration in zebrafish [24]. Additionally, miR-203a was reported to negatively regulate the expression of the aryl hydrocarbon receptor (AhR) in human hepatic and lung cells [27]. Given that miR-203a had a consistently decreased expression in ELS mahi-mahi and red drum following exposure to a variety of oil types and concentrations, with similarly predicted diseases and dysfunctional pathways, an inhibition of miR-203a by oil may lead to morphological, physiological, and behavioral-level effects in ELS fish.

The objective of this study was to examine if decreased levels of miR-203a during embryonic development is sufficient to exhibit oil-like alterations in visually-mediated and neuro-cardiotoxic pathways in ELS zebrafish. Phenanthrene was used as a positive control due to its similarity in toxic response with weathered crude oil [4]. Gene expression profiles and downstream morphological, physiological, and behavioral-level responses were compared between fish exposed to Phe and fish injected with the miR-203a inhibitor. We hypothesized that (1) embryos injected with a miR-203a inhibitor would exhibit impaired eye formation and visual effects, (2) injected zebrafish would exhibit altered cardiac function and fin development and, (3) miR-203a inhibitor-injected fish would exhibit similar effects as phenanthrene-exposed fish, which would be representative of crude oil exposure.

## 2 Materials and methods

### 2.1 Fish husbandry

Adult wild-type zebrafish were housed in static, 40-L glass aquaria containing dechlorinated tap water maintained at 28 ± 1 °C under a 14:10 h light:dark photoperiod. Adults were fed GEMMA Micro 300 (Skretting, ME, USA) ad libitum daily. Water changes were conducted daily following feeding and water quality maintained. Adult male and female zebrafish were transferred into 3 L tanks with in-tank breeding traps overnight at a 1:1 sex ratio to obtain embryos the following morning. Immediately following spawning events, embryos were collected and placed into E3 media (5 mM NaCl, 0.17 mM KCl, 0.33 mM CaCl_2_, and 0.33 mM MgSO_4_) where embryos were staged according to previous methods [28]. All experiments were performed ethically and in accordance with the Institutional Animal Care and use Committee (AUP# 20190003) approved by the University of California, Riverside IACUC committee.

### 2.2 miRNA microinjections

Embryonic zebrafish were injected at the one-to-eight cell stage (cleavage-stage) with 225 moles of a miR-203a inhibitor or negative control (nc-miR) with an Eppendorf Injectman N12 and FemtoJet 4x, as described previously [29]. A nc-miR was used to generate a baseline for comparing against miR-203a inhibitor treatments in order to account for any non-treatment-induced effects due to the microinjection process, as injection of nc-miR alone does not produce known effects to miRNA function or downstream mRNA expression profiles. Lyophilized miR-203a inhibitor (mirVana miRNA inhibitor has-miR-203a-3p, cat #4464084, assay ID MH10152, mature miRNA sequence: 5′-GUGAAAUGUUUAGGACCACUAG-3′) and nc-miR (Negative Control #1, cat #4464058) were obtained from Thermo Fisher Scientific (Waltham, MA, USA), reconstituted in molecular-grade water, and stored at − 20 °C. Working concentrations of the miR-203a inhibitor and nc-miR (75 µM) were prepared immediately prior to injections by diluting stock solutions (250 µM) in molecular-grade water. Injection volume and concentration levels were based on initial range-finding experiments and did not induce > 30% mortality.

### 2.3 Phenanthrene exposure

Phe stock solutions were prepared by dissolving Phe (98% purity, Sigma Aldrich) in 100% dimethyl sulfoxide (DMSO). E3 water was spiked with DMSO or Phe stock to produce a DMSO vehicle control (0.02% DMSO) or a 3.5 µM Phe exposure solution. Vehicle controls and Phe treatments were conducted in triplicate with 100 embryos per 300 mL glass beaker in a 27 ± 1 °C incubator under a 14:10 light:dark photoperiod. Embryos were collected immediately following spawning events, staged, and sorted prior to waterborne exposures. Embryos (1–1.25 hpf) were exposed for 7, 48, 72, or 120 h post fertilization (hpf), depending on the endpoint assessed, with > 90% exposure renewal frequency conducted daily.

### 2.4 Morphometric and physiological assessment

Heart rate (beats per minute) was measured in 48 hpf embryos (n = 10 per treatment) by recording the total number of heartbeats within a 20 s video clip and multiplying the values by 3. Body length and caudal fin development were determined from 72 hpf larvae (n = 10 per treatment). Deformity rate (cardiac edema, eye development, spinal deformity) was assessed in 72 hpf larvae. Lens and eye size were measured in 120 hpf larvae (n = 15 per treatment). All larvae were imaged under a Leica MZ10 F stereomicroscope (Leica Microsystems, Buffalo Grove, IL) with a DMC2900 camera. Measurements were assessed using ImageJ (version 1.52a; [30]).

### 2.5 Optokinetic response assay

An optokinetic response (OKR) assay was conducted with 120-hpf larvae (n = 15 per treatment) as previously described [6]. Briefly, larvae were placed in 3% methylcellulose to prevent side-to-side movement within a 35 mm Petri dish (Brockerhoff et al., [31]; Brockerhoff [32]). Larvae were positioned equidistant to the side of a circular drum with black and white alternating vertical stripes, with the stripes subtending a 9° angle to the larvae and rotated at 6 rpm by a driven motor (Magnuson et al., [33]; [6]). The outer drum was rotated in clockwise and counterclockwise directions in 1-min increments. Larval eye movements (saccades) were recorded for clockwise and counterclockwise directions, with the mean value recorded during analysis.

### 2.6 miRNA extractions and qPCR

To confirm miR-203a inhibitor injections and the effects of miR-203a inhibition and Phe-exposure on downstream mRNA gene expression, quantitative polymerase chain reaction (qPCR) was conducted on microinjected embryos at 7 and 72 hpf. A 7 hpf timepoint was chosen to determine expression during a critical developmental timepoint for morphogenesis, 50–75% epiboly. A total of 20 embryos (7 hpf) and larvae (72 hpf) were pooled in triplicate (n = 3 in each treatment at each developmental timepoint), flash frozen in liquid nitrogen, and stored at − 80 °C until miRNA extractions. Zebrafish were homogenized with a handheld homogenizer (Omni International, Kennesaw, GA) and a miRNeasy mini kit (Qiagen, Valencia, CA, USA) used for extracting total RNA, per the manufacturer’s instructions. A Nanodrop 2000c (Thermo Fisher Scientific, Waltham, MA) was used for measuring RNA concentrations. A miScript II RT Kit (Qiagen) was used for synthesizing cDNA and miScript SYBR Green (Qiagen) was used for qPCR. RNU6 was used as a normalizing gene for miR-203a inhibitor expression. All qPCR reactions were conducted in triplicate on a Bio-Rad CFX Connect per the manufacturer’s instructions. The subset of genes chosen for gene expression analysis were based on the role miR-203a has in targeting and regulating *klf4* (Xu et al., [34]), *ahr2* [27], *vegfa* [35], *neurod1* and *crx* [20]. *pde6h* was chosen because it is regulated in part by crx (Ruzycki et al., [36]; Corbo et al., [37]) and acts along the phototransduction pathway, which has previously been reported to be dysregulated following oil exposure [6]. Relative mRNA expression of genes was determined by the 2^-ΔΔCt^ method (Livak and Schmittgen 2001). Elongation factor 1 alpha (*ef1a*) was used as the housekeeping gene for mRNA gene expression, as previously conducted [38]. miR-203a inhibitor and RNU6 primers were obtained from Qiagen miRNA primer assay line. Primer sequences for mRNA qPCR are provided in Table S1.

### 2.7 Statistics

SPSS (version 27) was used for performing statistical analyses. A Shapiro-Wilk and Levene’s test were used to test for normality and homogeneity of variance, respectively. A one-way ANOVA, with a Tukey’s post-hoc, was used to compare miRNA and mRNA gene expression between treatment groups. Independent sample T-tests were conducted to determine mean differences between morphometric, physiological, and optokinetic response assay assessments. A p-value < 0.05 was used to denote statistically significant differences.

## 3 Results

### 3.1 Confirmation of miR-203a inhibition

**Fig. 1:**
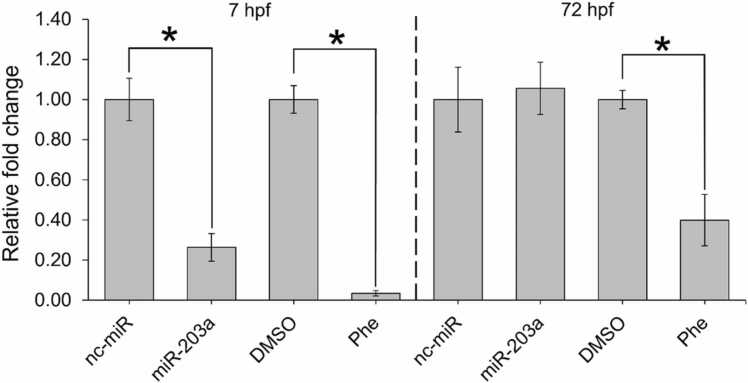
qPCR confirmation of miR-203a expression in 7 and 72 hpf zebrafish, relative to controls. (One-way ANOVA, Tukey’s post-hoc; mean ± SEM; * p < 0.05), n = 3 treatment replicates.

To confirm that miR-203a was inhibited, the expression of miR-203a was determined in 7 and 72 hpf microinjected embryos and Phe-exposed treatments. There was a significant decrease in miR-203a expression in embryos injected with the miR-203a inhibitor by 7 hpf (p = 0.004), though there was no significant change in expression by 72 hpf (p = 0.802; Fig. 1), relative to nc-miR treatments. Phe-treated zebrafish had a significant decrease in miR-203a expression in 7 hpf (p < 0.001) and 72 hpf (p = 0.012) embryos, relative to DMSO controls (Fig. 1).

### 3.2 Morphometric and physiological assessment

**Fig. 2:**
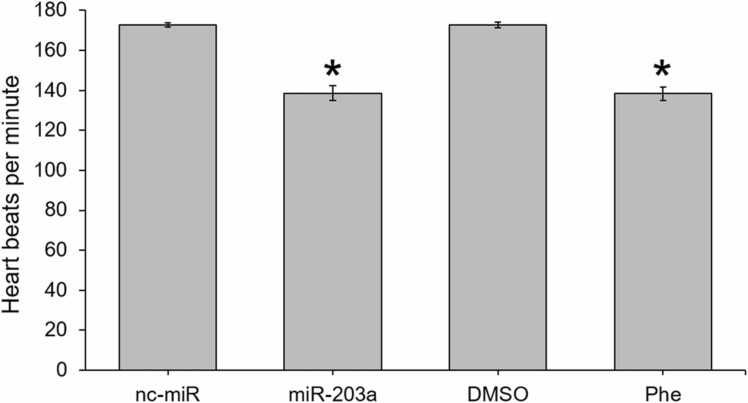
The effect of miR-203a inhibitor and phenenthrene treatment on heart rate (beats per minute) in 48 hpf zebrafish relative to controls. (Student’s t-test; mean ± SEM; * p < 0.05), n = 10 per treatment.

miR-203a inhibitor-injected and Phe-treated embryos had significantly reduced heart rates by 48 hpf, relative to nc-miR and DMSO controls (p < 0.001 and p < 0.001, respectively; Fig. 2). There was an overall increase in the rate of deformities (pericardial edema, eye deformity, and spinal deformities) in miR-203a inhibitor-injected and Phe-treated larvae by 72 hpf (Fig. S1), with a significantly decreased body length (p = 0.002), fin height (p < 0.001) and fin depth (p < 0.001) in zebrafish treated with Phe.

**Fig. 3:**
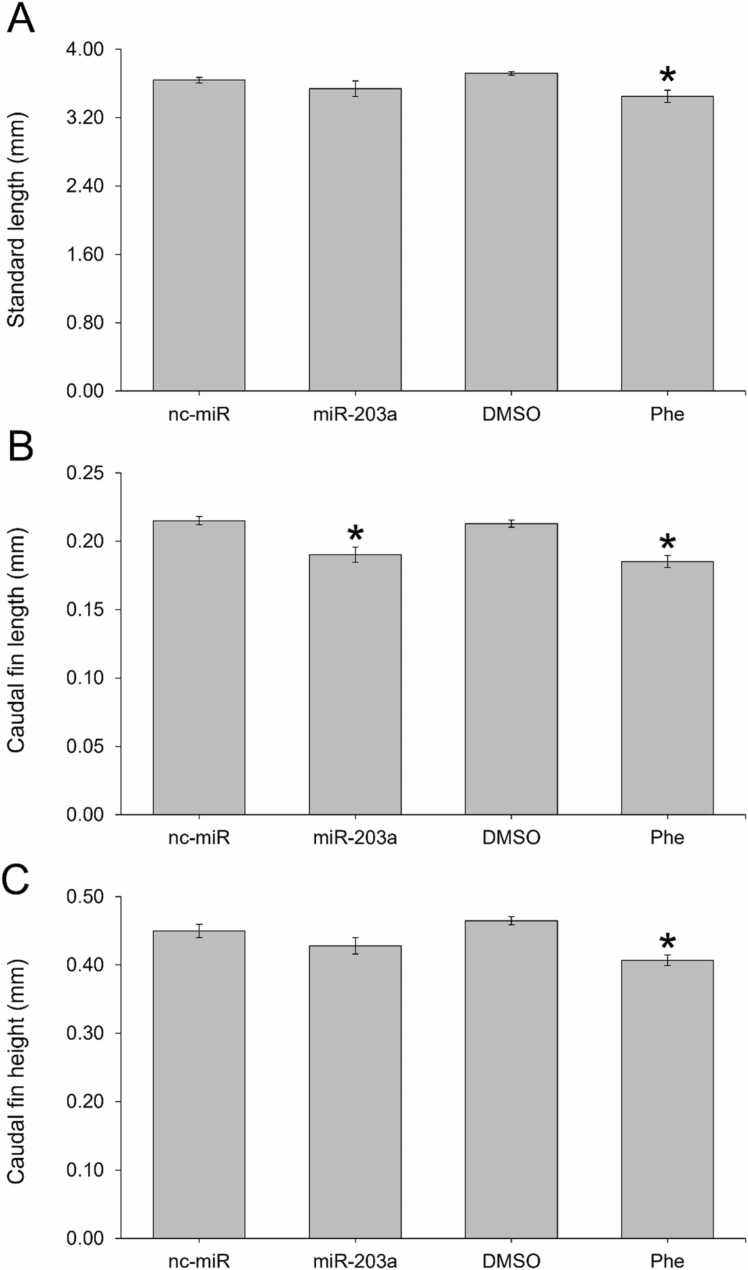
The effect of miR-203a inhibitor and phenenthrene treatment on the (A) standard length, (B) caudal fin length, and (C) caudal fin height in 72 hpf zebrafish relative to controls. (Student’s t-test; mean ± SEM; * p < 0.05), n = 10 per treatment.

**Fig. 4:**
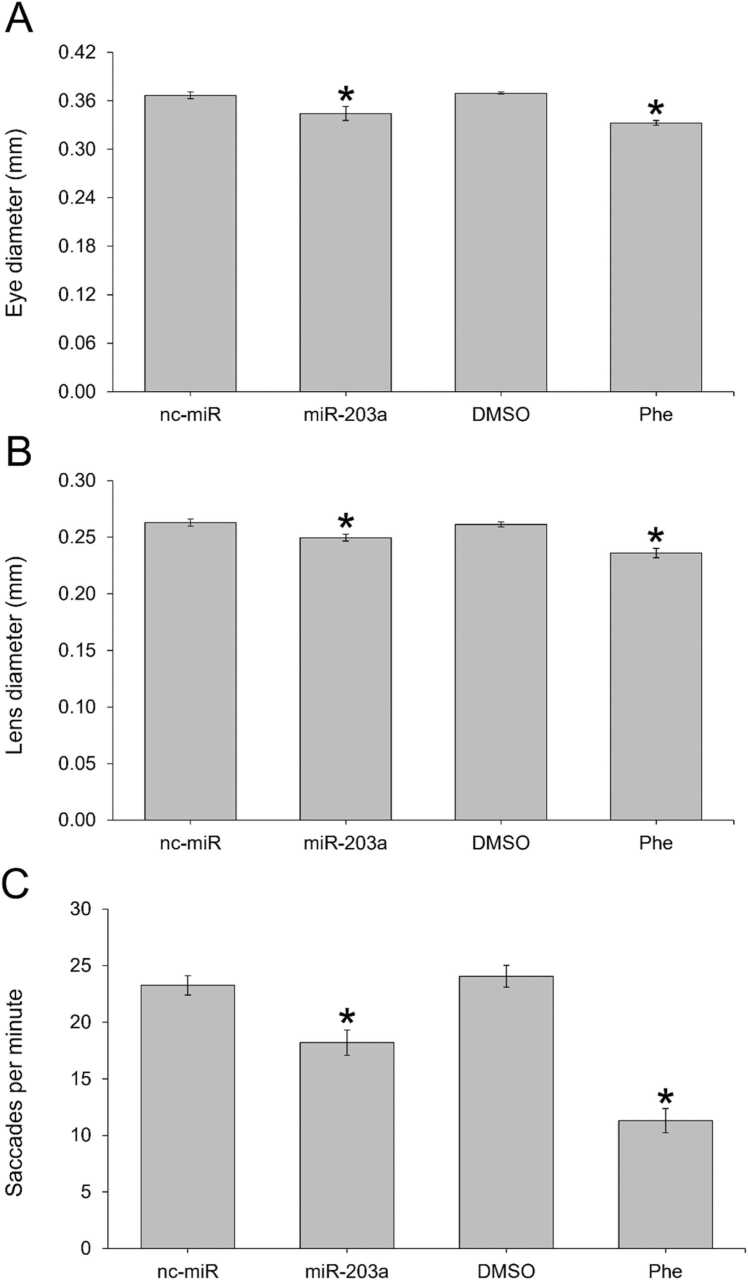
The effect of miR-203a inhibitor and phenenthrene treatment on (A) eye diameter, (B) lens diameter, and (C) eye saccades per minute in 120 hpf zebrafish relative to controls. (Student’s t-test; mean ± SEM; * p < 0.05), n = 15 per treatment.

miR-203a inhibitor-injected embryos had a significantly reduced fin length (p < 0.001), though not fin width (p = 0.175) by 72 hpf (Fig. 3). There was a significantly reduced lens diameter in miR-203a inhibitor-injected (p = 0.005) and Phe-treated 120 hpf larvae (p < 0.001) and reduced eye diameter in miR-203a inhibitor-injected (p = 0.025) and Phe-treated larvae (p < 0.001) (Fig. 4).

### 3.3 Optokinetic response- eye saccades

By 120 hpf, miR-203a inhibitor-injected and Phe-exposed zebrafish had a significantly reduced number of eye saccades relative to controls (p = 0.001 and p < 0.001; Fig. 4). miR-203a inhibitor-injected embryos had an average of 18.20 ± 1.11 eye saccades per minute (saccades/min) relative to nc-miR injected embryos, which had an average of 23.27 ± 0.87 saccades/min. Phe-treated embryos had an average of 11.30 ± 1.08 saccades/min relative to DMSO controls with an average of 24.07 ± 0.96 saccades/min.

### 3.4 Gene expression

**Fig. 5:**
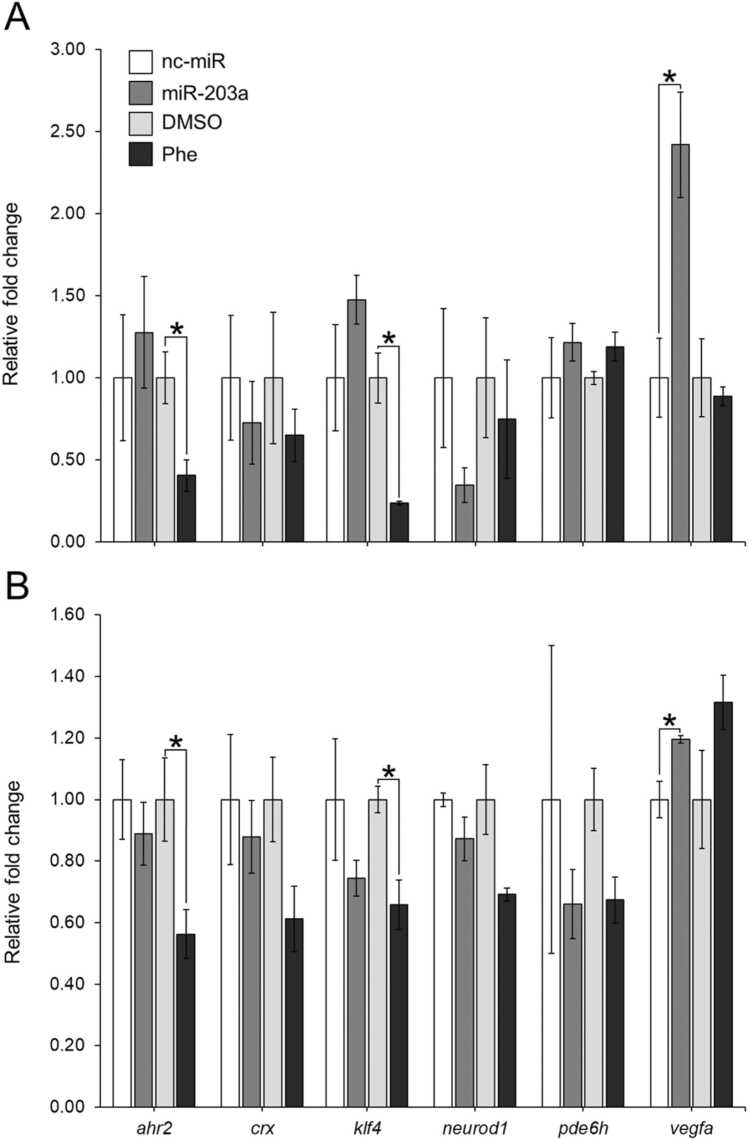
The effect of miR-203a inhibitor and phenenthrene treatment on mRNA gene expression in (A) 7 hpf and (B) 72 hpf zebrafish relative to nc-miR and DMSO controls, respectively. (One-way ANOVA, Tukey’s post-hoc; mean ± SEM; * p < 0.05), n = 3 pooled replicates (20 embryos (7 hpf) and larvae (72 hpf)) from each treatment.

By 7 hpf, miR-203a inhibitor-injected embryos had a significant increase in *vegfa* mRNA expression (p = 0.024), though no significant alteration in the expression of *ahr2*, *crx*, *klf4*, *neurod1*, or *pde6h* at 7 hpf. There was a significant reduction in *ahr2* (p = 0.033) and *klf4* (p = 0.008) mRNA gene expression in Phe-treated embryos at 7 hpf. By 72 hpf, miR-203a inhibitor-injected zebrafish had significantly upregulated *vegfa* (p = 0.032) mRNA expression. In Phe-treated zebrafish, significant reductions of *ahr2* (p = 0.049) and *klf4* (p = 0.020) mRNA expression were still present at 72 hpf, though there was no significant alteration in the mRNA expression of other genes examined (Fig. 5).

## 4 Discussion

Recent studies have reported significant changes in miRNA and mRNA expression profiles which predicted impaired morphology and function in ELS fish after treatment with crude oil [12], [10], [11]. In silico software evaluating overlays of these data have provided novel predictions of altered apical events based on molecular-level changes and have allowed the derivation of hypothesis-driven research to reduce uncertainty in risk assessments of oil-derived compounds. Suppression of miR-203a was observed in mahi-mahi and red drum following treatment to several forms and concentrations of oil, which was associated with enhanced expression of mRNAs that predicted alterations of neurological, cardiovascular, ophthalmic, and skeletal/muscular disorders [12], [10]. To determine if miR-203a inhibition would produce oil-like effects to visually-mediated or cardiac pathways, zebrafish embryos were injected with an miR-203a inhibitor and a previously characterized model PAH in oil, phenanthrene, and assessed for molecular, physiological, morphological, and behavioral-level effects throughout early stages of development.

Zebrafish embryos injected with a miR-203a inhibitor and those exposed to the positive control, Phe, had reduced heart rates by 48 hpf. Decreased miR-203a abundance was predicted to induce cardiovascular disease in ELS mahi-mahi exposed to DWH oil [10], [11] and significantly reduce heart rate following oil treatment [10]. Additionally, ELS red drum exposed to DWH oil were predicted to have altered heart valve morphogenesis, which was associated with decreased miR-203a expression [12]. Decreased levels of miR-203a have been shown to be associated with chronic human heart failure [39] and correlated with heart hypertrophy and cardiac fibrosis [40].

Treatment with Phe caused a similar down-regulation of miR-203a expression in 7 and 72 hpf zebrafish. Though it is unclear how PAHs, such as Phe, impair miR-203a in fish, previous studies evaluating the regulation of miR-203a indicated that alterations in CpG methylation on the miR-203a promoter may be involved [41]. Phe has been shown to increase DNA hypermethylation [42], [43] and reduced the expression of miR-133a in H9C2 cells treated with 50 nM Phe through an increase in methylation of DNA at CpG sites at the miR-133a locus [44]. Other PAHs found in oil (including benzo(a)pyrene, fluoranthene, and pyrene) also caused hypermethylation of DNA [45] and altered miRNAs which may influence epigenetic regulation [46]. Additional studies are needed to confirm whether methylation of the miR-203a promoter or other signaling targets are altered by Phe or other oil-derived PAHs.

Reduced caudal fin length was seen in miR-203a inhibitor-injected zebrafish by 72 hpf. Phe-exposed treatments also exhibited reduced caudal fin length and reduced fin height. Similarly, reduced caudal fin growth was reported in ELS fish following oil treatment [5], [47]. In adult zebrafish, miR-203a was reported to target Lef1, a Wnt signaling transcription factor, which was necessary for caudal fin regeneration to occur [24]. An inhibition of miR-203a increased Lef1 protein levels and induced fin growth [24]. Though miR-203a inhibition is an important factor for fin regeneration to occur, the inhibition of miR-133 is subsequently required (Yin et al., [48]; Tal et al., [49]), suggesting that a downregulation in both miR-203a and miR-133 is necessary for fin growth. Reduction in fin height and width observed in this study is consistent with a regulatory role of miR-203a in this developmental process.

miR-203a inhibitor-injected embryos had reduced lens and eye diameters by 120 hpf, with subsequent reductions in the number of eye movements (saccades), determined by using an OKR behavioral assay. Phe-exposed embryos also had reduced lens and eye diameters and exhibited a reduced OKR. Reduced eye saccades were previously observed in ELS zebrafish following DWH crude oil exposure [6], further validating that an inhibition of miR-203a may induce similar morphological and behavioral-level responses following oil exposure. miR-203a downregulates the proliferation of progenitor cells in the zebrafish retina and targets several retina-specific genes during various stages of photoreceptor development [23], [20]. mRNA expression of *crx*, *neurod1*, and *pde6h* had negative trends in 72 hpf larvae that were embryonically injected with the miR-203a inhibitor or exposed to Phe. Similarly, *crx* and *pde6h* mRNA expression was downregulated in ELS zebrafish exposed to DWH crude oil [6].

Treatment of human somatic stem cells with miR-203a was previously shown to decrease *crx* and *neurod1* expression during the photoreceptor precursor developmental stage [20]. However, o*tx2*, an important component of the Otx homeobox family that control retinal photoreceptor cell development [50], is upstream of *crx* [50], [20] and regulated by miR-410 [51]. There are likely interactions between several miRNAs that influence retinal cell function [21], with additional interrelationships between *let7* and miR-203a levels shown to influence Müller glial cell proliferation through the suppression of *pax6b* [52]. Expression of *pax6* was significantly decreased in the retinas of 72 hpf zebrafish treated with 0.2 µM Phe [53], indicating regulation may be altered through upstream modifications of miR-203a. Studies looking at the expression of eye-specific genes and the influence of individual PAHs, relative to mixtures, are limited and additional studies are warranted to better characterize associated mechanisms, with a focus on miRNA regulation.

Vegfa, an important activator during neovascularization [55], [54], [56], is a downstream target of miR-203a-3p and involved in the regulation of miR-203–3p during retinal angiogenesis [35]. There was a significant induction of *vegfa* mRNA expression in embryos injected with the miR-203a inhibitor by 7 and 72 hpf, and a positive trend in Phe-exposed zebrafish by 72 hpf. The transcription factor, klf4, binds to the VEGF promoter, induces the expression of *vegf*, and promotes downstream angiogenesis of the microvasculature of endothelial cells in the retina [57], which could reduce gas and nutrient exchange and impair proper eye development. *Klf4* expression was significantly decreased in 7 and 72 hpf zebrafish that were treated with Phe and a downward trend was observed in 72 hpf larvae microinjected with the miR-203a inhibitor. Platelet-derived growth factor-BB (PDGF-BB) can increase KLF4 promotor activity, allowing it to bind to the VEGF promotor, which transcriptionally activates the mRNA expression of *vegfa*. Additionally, PDGF-BB can bind to VEGF itself, directly regulating its expression [58], [57]. It is possible that there was a transcriptional shift in PDGF-BB between 7 and 72 hpf that induced *vegfa* expression, acting through different pathways, by increasing the promotor activity in KLF4 during embryonic stages while acting directly on VEGF when at the larval stage. Further experiments are warranted to understand the relationship between PDGF-BB, KLF4, and VEGF during early life stage development in zebrafish. Considering the role Klf4 has in eye lens maturation [60], [61], [59], a downregulation of *klf4* mRNA expression due to an inhibition of miR-203a likely contributed to microphthalmia in treated fish, which could have implications to visual acuity and foraging behavior in ELS fish [62].

Embryos treated with Phe had significantly decreased *ahr2* mRNA expression by 7 and 72 hpf, whereas miR-203a inhibitor-injected zebrafish did not have a significantly altered expression at either time point. miR-203a expression was reported to negatively regulate AhR expression in human hepatic and lung cells treated with 100 nM miR-203a [27]. The differences in *ahr2* expression profiles between Phe and miR-203a inhibitor treatments may be reflective of the dose of the miR-203a inhibitor used, which was lower than what was previously used to expose hepatic and lung cells with (100 nM) for 24 h to exhibit an increased *ahr* expression [27]. Alternatively, differences in miR expression and regulation may also occur in immortalized cell culture relative to developing embryos of multiple cell types. Although Phe-induced cardiotoxicity has been shown to be mediated through a non-AhR pathway, Phe selectively binds to AhR2 in zebrafish [4]. The affinity of miR-203a to AhR2 versus AhR1 may also be different, however, explaining the variability between Phe and injection treatments, though this would need to be further investigated.

## 5 Conclusions

miR-203a plays a critical role in the proper development and function of fish, with the inhibition of miR-203a impairing cardiac function, eye and fin morphology, and visually-mediated responses in ELS fish, paralleling oil-induced effects. This suggests that miR-203a may be involved in the altered regulation of genes that mediate cardiovascular, ophthalmic, and/or skeletal/muscular disorders and could be used to better understand the mechanisms of impaired heart, vision, or fin development in oil-exposed fish.

## CRediT authorship contribution statement

**Jason Magnuson:** Conceptualization, Investigation, Formal analysis, Visualization, Writing − original draft. **Le Qian:** Investigation, Formal analysis, Validation, Visualization, Writing − review & editing. **Victoria McGruer:** Investigation, Formal analysis, Writing − review & editing. **Vanessa Cheng:** Investigation, Writing − review & editing. **David Volz:** Resources, Writing − review & editing. **Daniel Schlenk:** Conceptualization, Methodology, Writing − review & editing, Supervision, Project administration, Funding acquisition.

## Funding

This work was supported by funding from the UCR/AES Resource Allocation Program and 10.13039/501100004543China Scholarship Council10.13039/501100004543.

## Declaration of Competing Interest

The authors declare that they have no known competing financial interests or personal relationships that could have appeared to influence the work reported in this paper.
