# Supplementary material for: Relationship between miR-203a inhibition and oil-induced toxicity in early life stage zebrafish (Danio rerio)
Source: Toxicol Rep. 2022 Mar 7;9:373–81. doi: 10.1016/j.toxrep.2022.03.006 (PMC8914477; doi:10.1016/j.toxrep.2022.03.006)
Supplement: Supplementary file 1 — Supplementary material [file mmc1.docx]

Relationship between miR-203a inhibition and oil-induced toxicity in early life stage zebrafish (*Danio rerio*)

Jason T. Magnuson^a,*^, Le Qian^b,c,*^, Victoria McGruer^a^, Vanessa Cheng^a^, David C. Volz^a^, Daniel Schlenk^a,d^

^a^Department of Environmental Sciences, University of California, Riverside, CA, USA

^b^College of Sciences, China Agricultural University, Beijing, China

^c^College of Horticulture and Plant Protection, Henan University of Science and Technology,

Luoyang, China

^d^Institute of Environmental Health, College of Environmental and Resource Sciences, Zhejiang

University, Hangzhou 310058, China

Table S1. Primer sequences used for qPCR analysis.

| Gene | Direction | Primer sequence (5’-3’) | Amplicon length (bp) | NCBI accession |
| --- | --- | --- | --- | --- |
| *ahr2* | Forward  Reverse | GTCCCACACTGGATGTTGCTG  CGCTTTGTCAATGGTAAACTGGGA | 167 | NM_131264.1 |
| *crx* | Forward  Reverse | CGTCGTTGGGCTTCAGTT  CTTCACGCATCTTTCCTTCC | 166 | NM_152940.1 |
| *klf4* | Forward  Reverse | GATAGCATGGCACTGAGCGGAAC  ACATCTCCTCCTTCCATCTTGAACCG | 124 | NM_001113483.1 |
| *neurod1* | Forward  Reverse | GCTCTCACATCTTTCACGTCAAGCC  GTCAAATGAGGGACTGGTGCAG | 98 | NM_130978.2 |
| *pde6h* | Forward  Reverse | CAGAAGCTCCAGCACAGCAC  GATGTCTGTGCCGAGACCCTC | 179 | NM_200785.1 |
| *vegfa* | Forward  Reverse | TGTAATGATGAGGCGCTCGAA  AGGCTCACAGTGGTTTTCTT | 180 | NM_131408.3 |
| *ef1a* | Forward  Reverse | ATACATCAAGAAGATCGGCTACAA  CCACAGGTACAGTTCCAATAC | 266 | FJ915061.1 |


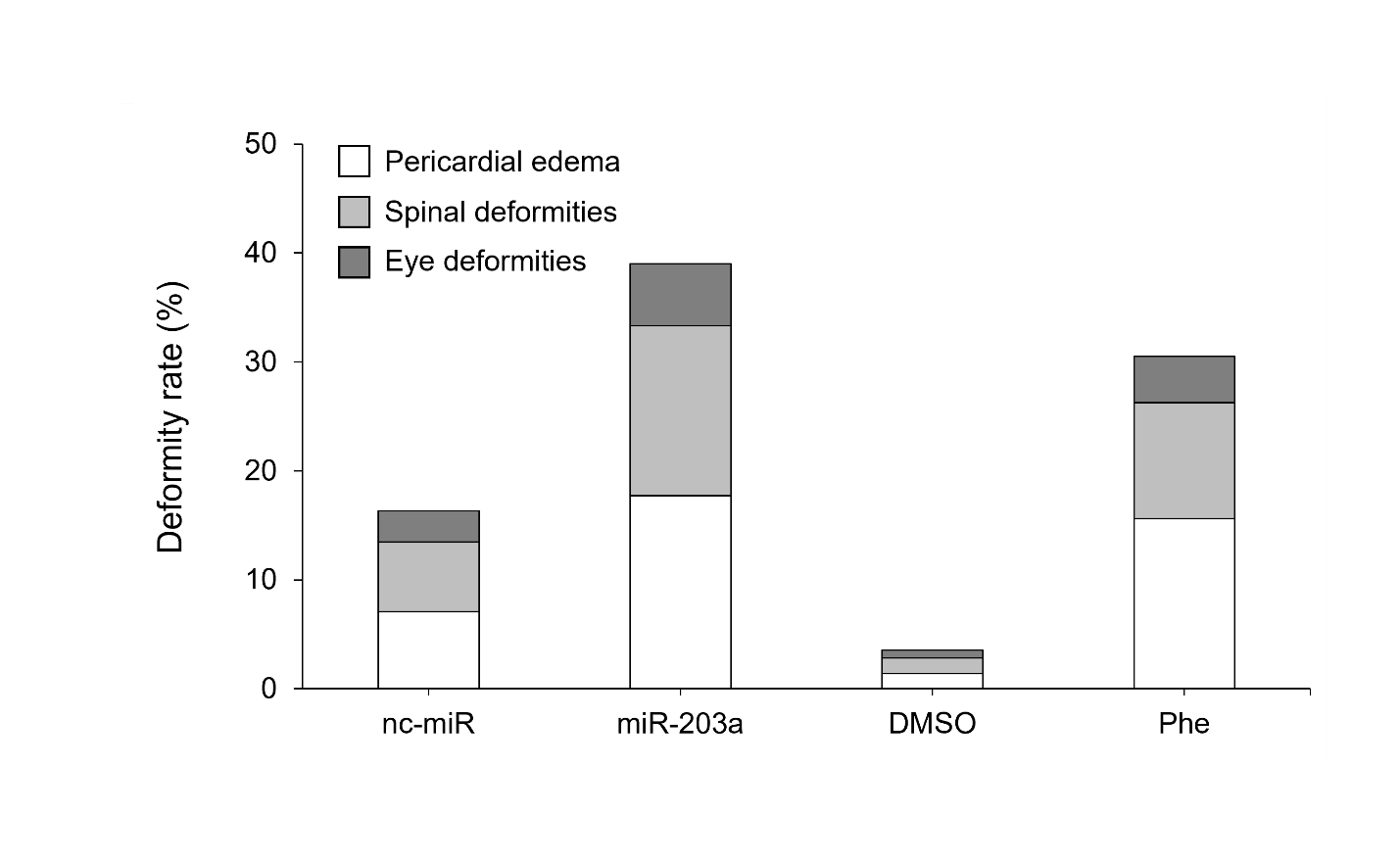


Figure S1. Rate of deformities (%) in miR-203a inhibitor injected and phenanthrene

exposed larvae by 72 hpf. n=140-142 per treatment.
